# Compromised Neurotrophic and Angiogenic Regenerative Capability during Tendon Healing in a Rat Model of Type-II Diabetes

**DOI:** 10.1371/journal.pone.0170748

**Published:** 2017-01-25

**Authors:** Aisha S. Ahmed, Jian Li, Alim M. D. Abdul, Mahmood Ahmed, Claes-Göran Östenson, Paul T. Salo, Carolyn Hewitt, David A. Hart, Paul W. Ackermann

**Affiliations:** 1 Karolinska Institutet, Department of Clinical Neuroscience, Stockholm, Sweden; 2 Karolinska Institutet, Department of Molecular Medicine and Surgery, Karolinska University Hospital, Solna, Stockholm, Sweden; 3 Karolinska Institutet, Department of Neurobiology, Care Sciences and Society, Center for Family and Community Medicine (CeFAM), Huddinge, Sweden; 4 McCaig Institute for Bone & Joint Health, University of Calgary, Calgary, AB, Canada; Universita degli Studi di Parma, ITALY

## Abstract

Metabolic diseases such as diabetes mellitus type-II (DM-II) may increase the risk of suffering painful connective tissue disorders and tendon ruptures. The pathomechanisms, however, by which diabetes adversely affects connective tissue matrix metabolism and regeneration, still need better definition. Our aim was to study the effect of DM-II on expressional changes of neuro- and angiotrophic mediators and receptors in intact and healing Achilles tendon. The right Achilles tendon was transected in 5 male DM-II Goto-Kakizaki (GK) and 4 age-matched Wistar control rats. The left Achilles tendons were left intact. At week 2 post-injury, NGF, BDNF, TSP, and receptors TrkA, TrkB and Nk1 gene expression was studied by quantitative RT-PCR (qRT-PCR) and their protein distribution by immunohistochemistry in intact and injured tendons. The expression of tendon-related markers, Scleraxis (SCX) and Tenomodulin (TNMD), was evaluated by qRT-PCR in intact and injured tendons. Injured tendons of diabetic GK rats exhibited significantly down-regulated *Ngf* and *Tsp1* mRNA and corresponding protein levels, and down-regulated *Trka* gene expression compared to injured Wistar controls. Intact tendons of DM-II GK rats displayed reduced mRNA levels for *Ngf*, *Tsp1* and *Trkb* compared to corresponding intact non-diabetic tendons. Up-regulated *Scx* and *Tnmd* gene expression was observed in injured tendons of normal and diabetic GK rats compared to intact Wistar controls. However, these molecules were not up-regulated in injured DM-II GK rats compared to their corresponding controls. Our results suggest that DM-II has detrimental effects on neuro- and angiotrophic pathways, and such effects may reflect the compromised repair seen in diabetic Achilles tendon. Thus, novel approaches for regeneration of injured, including tendinopathic, and surgically repaired diabetic tendons may include therapeutic molecular modulation of neurotrophic pathways such as NGF and its receptors.

## Introduction

Patients with metabolic disorders such as type-2 diabetes mellitus (DM) are at increased risk of suffering various musculoskeletal disorders [1,2]. Painful connective tissue diseases associated with DM, such as osteoarthritis, capsulitis, tendinopathy and tendon ruptures can result in considerable disability due to compromised regenerative capability. The underlying neurotrophic and angiotrophic pathways, which are altered in connective tissue homeostasis and regeneration of metabolic diseases, however, are far from completely understood [3–5].

Connective tissues are comprised of cells (mainly fibroblasts) and a tissue-specialized extracellular matrix (ECM) containing proteoglycans, polysaccharides, and collagen. The homeostasis and regenerative capability of the ECM is vital for mechanical integrity, tissue growth and wound healing. Diabetes, however, can adversely affect the properties of the native ECM [2, 6]. Patients with diabetes often exhibit delayed and/or defective tissue healing, associated with impaired formation of a collagen matrix, compromised angiogenesis and hampered neuronal function [4, 7].

Neuronal growth factors have demonstrated essential functions in both re-innervation and angiogenesis involved in connective tissue homeostasis and regeneration. Neurotrophins found in tendon tissue include nerve growth factor (NGF) and brain-derived neurotrophic factor (BDNF), both of which are essential for wound healing. Moreover, a metabotrophic role of NGF and BDNF has recently been implicated in the pathogenesis of diabetes related disorders [8]. Notably, patients with diabetic neuropathy have lower serum NGF levels than controls, and the decrease in NGF is reported to be proportional to decreases in a patients’ nerve conduction velocity [9]. NGF and BDNF act via their respective receptors TrkA and TrkB, which have been detected in tendon [10]. It has also been reported that NGF can drive the up-regulation of expression of sensory neuropeptides such as Substance P (SP) [11]. SP has, via its receptor neurokinin 1 (NK1), been found to stimulate recruitment of stem cells to injury sites [12] and promote wound healing in diabetes. NGF and NK1 stimulation has been demonstrated to promote connective tissue repair, in part by enhancing angiogenesis [13].

In addition, angiogenesis is critical to healing of dense, hypovascular connective tissues such as tendon, but needs to be tightly regulated to avoid unnecessary disruption and weakening of the collagen structure. Interestingly, it has been reported that thrombospondin (TSP), a matrix-associated factor, inhibits angiogenesis by specifically suppressing NK1 stimulation [14].

In the present study, we hypothesized that abnormal expression of neurotrophic and angiotrophic factors may contribute to impaired connective tissue homeostasis and repair associated with diabetes. The specific aim of the present study was to assess the expressional changes of the above mentioned neuro- and angiotrophic genes and proteins in intact and healing connective tendon tissue of rats with type 2 diabetes mellitus, compared to healing in a non-diabetic strain of rat. Further, tendon-related markers, Scleraxis (*Scx*) and Tenomodulin (*Tnmd*) gene expression were studied in intact and healing tendons of type 2 diabetes and non-diabetic rats. We used the Goto–Kakizaki (GK) rat, which is a well-established non-obese type-II diabetes model produced by selective breeding from non- diabetic Wistar rats with glucose intolerance as a selection index [15]. GK rats develop elevated blood glucose levels and exhibit reduced nerve conduction velocity (NCV) reflecting the presence of neuropathy which is a common complication of diabetes as reported previously [16]. These rats also demonstrate impaired connective tissue healing of the injured Achilles tendon [6, 19].

## Materials and Methods

### Study design

This study included five 12-month old Goto-Kakizaki (GK) male rats from the breeding colony at the Karolinska University Hospital and four age- and sex-matched control Wistar rats. All animals were housed at 210C in a 12 hour light/dark cycle with pellets and water ad lib according to the Karolinska Institute protocol. All experiments were approved by the Committee for Animal Research and Ethics Stockholm North and conducted in accordance with the Institute’s protocols.

### Surgery

All rats were anaesthetized by a single injection of a mixture of ¼ Midazolam® (5mg/ml, Pharma Hameln, Germany) and ¼ Hypnorm® (Janssen Pharmaceutica, Belgium) in sterile water (2ml/kg bw, s.c). The Achilles and plantaris tendons of the right legs were exposed through a 1-cm midline posterior longitudinal incision under sterile conditions. By using a blunt instrument, both tendons were fully ruptured in the mid part, approximately 0.5 cm from the calcaneal insertion. The tendons were left unsutured and the skin was closed with two sutures of 5/0 nylon monofilament (2x2, Ethilon®II, Ethicon, USA). Postoperatively, all animals were allowed free cage activity and Temgesic (buprenorphine 0.2 mg/kg) was administered as required.

### Dissection

At two weeks post rupture, rats were anesthetised with sodium pentobarbitone (60 mg/kg, intraperitoneal) and euthanatized. The right (ruptured) and left (intact control) Achilles and plantaris tendons were removed along with the gastrocnemious muscle and the calcaneal bone and sagittally divided into medial and lateral segments. The medial segments were immediately frozen in liquid nitrogen and kept in -70°C until RNA isolation and qRT-PCR assessment. The lateral segments were immediately soaked in Zamboni´s fixative consisting of 4% paraformaldehyde in 0.2 mol/l Sörensen phosphate buffer, pH 7.3, containing 0.2% picric acid at 4°C for two days. It was ensured that only tissue from the rupture site (i.e. callus) was taken from the ruptured tendons.

### Real-time quantitative PCR

Frozen medial tendon tissues were homogenized by Mikro-dismembrator (B. Braun Biotech International, Germany) and dissolved in 2–3 volumes of Trizol reagent (Invitrogen Life Technologies Inc., USA). RNA was extracted and purified using the RNeasy^®^ MiniKit (Qiagen, USA) following the manufacturers protocol [18]. RNA integrity was assessed using the Agilent 2100 Bioanalyser (Agilent Technologies) according to the protocol of the company and loaded to the Eukaryote total RNA nano chip and processed. Quantification assays were performed to detect the relative mRNA expression of *Scx*, *Tnmd*, *Ngf*, *Bdnf*, *Tsp1*, *Trka*, *Trkb* and *Nk1* using techniques developed and optimized by our group as previously described [17, 18]. Briefly, total RNA (1ug) was reverse transcribed to generate single stranded cDNA using the Qiagen Omniscript RT kit (Qiagen Sciences, Gemantown MD, USA). PCR primers were synthesized and validated for the target molecules. BIO-RAD iQ SYBR Green Supermix (12.5 ul) (Bio-Rad, Hercules, CA), molecular biology water (3.5 ul), as well as forward and reverse primer (0.75 ul each) formed the PCR reaction mixture, using 7.5 ul RT for each reaction. All assays were performed in triplicate. The iCycler Thermal Cycler (Bio-Rad) was utilized during amplification and detection, validated through inspection of the melting curve (dF/dT vs temperature) for non-specific peaks. Levels of gene expression were normalized to *18s* rRNA levels. iCycler iQ Optical System Software version 3.0a (Bio-Rad) was used to quantify results.

### Immunohistochemistry

The lateral segments of the right (ruptured) and left (intact control) tendons dissected from Wistar and diabetic GK rats were fixed in Zamboni´s fixative consisting of 4% paraformaldehyde in 0.2 mol/l Sörensen phosphate buffer, pH 7.3, containing 0.2% picric acid at 4°C for two days. Tissues were then soaked in 20% sucrose in 0.1 mol/L Sörensen phosphate buffer, pH 7.2, containing sodium azide and bacitracin (Sigma Chemicals, St. Louis, MO, USA) until sectioning. Tendons were sectioned using a Leitz® 1720 cryostat (Ernst Leitz, Wetzlar, Germany) to a section thickness of 12 μm and mounted on SuperFrost/Plus slides. Three sections from each tendon at different depths (100 μm apart) were taken and stained for immunohistological assessment. Images were taken by a camera (DEI 750; Optronics Engineering, Goleto, CA) attached to microscope and saved in a computer.

Sections from right and left tendons from each group were stained with antibodies to NGF, BDNF, TSP1, Trk (recognises both TrkA and TrkB), and NK1. Non-specific binding was minimized by pre-incubating the sections in 5% normal goat serum for 30 minutes. The sections were then incubated overnight with specific antisera to NGF, BDNF, TSP1, Trk, NK1 (1:100, Santa Cruz Biotechnology, Santa Cruz, CA). Sections were rinsed in PBS (3x5 min) and incubated for 30 minutes at room temperature with secondary antibodies; goat anti-rabbit or donkey anti-goat (1:250, Vector Laboratories, Inc. Burlingame, CA, USA). The sections were then washed with PBS (3x5 min) and incubated with ABC reagent for 30 minutes at room temperature. This step was followed by application of diaminobenzidine (DAB) chromogen (Vector Laboratories, Inc. Burlingame, CA, USA) and counterstaining with Hematoxylin QS (Vector Laboratories, Inc. Burlingame, CA, USA). Sections were dehydrated with 70, 95, and then 99% ethanol. To confirm specificity of staining, control sections were stained with the primary antisera either omitted, or pre-adsorbed with the corresponding ligand peptides. Images were captured by a video camera (DEI 750; Optronics Engineering, Goleto, CA) attached to the microscope and stored in a computer for later analysis.

### Statistical analysis

The significance of the differences between injured and intact tendon groups was analysed by one way ANOVA followed by Fisher’s protected least significant difference test. The level of significance was set at *P* ≤ 0.05.

## Results

The presence of diabetes in GK rats was confirmed by fasting blood glucose levels, which were significantly higher in diabetic GK rats compared to Wistar controls, as reported previously [19].

### Gene expression patterns in diabetic connective tissue healing

RNA extracted from tendon of all four groups was of good quality with RNA integrity numbers ranging between 7.3 to 8.0 as reported previously [6]. At day 14 post-rupture, quantitative PCR analyses confirmed that all genes of interest (*Scx*, *Tnmd*, *Ngf*, *Bdnf*, *Tsp1*, *Trka*, *Trkb* and *Nk1*) were expressed at measurable levels in intact and healing tendons of both Wistar and GK rats.

#### *Scx*, *Tnmd* mRNA levels

**Fig 1:**
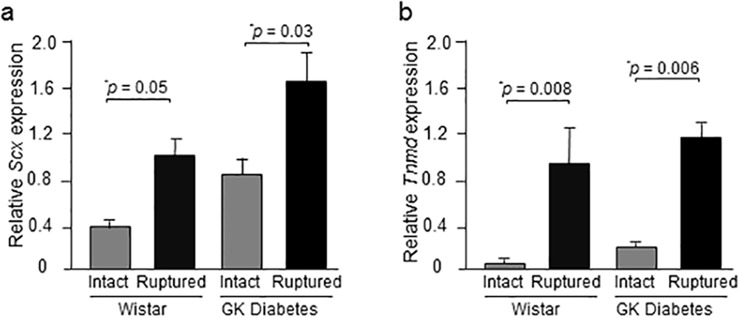
Relative expression of *Tnmd* and *Scx* mRNA in Achilles tendons. (a *Scx* and (b *Tnmd* gene expression in intact and ruptured Achilles tendons of Wistar and diabetic GK rats at day 14 post-rupture. Results are expressed as mean ± SEM with n = 4 in Wistar groups and n = 4 in diabetic GK groups (ANOVA followed by Fisher’s protected least significant difference test).

At 2 weeks post-injury, *Scx* and *Tnmd* gene expression was quantified in intact and healing tendons of both Wistar and diabetic GK rats. The ruptured tendons of Wistar rats exhibited 1.7- and 16-fold increase in *Scx* [p = 0.05] and *Tnmd* [p = 0.008] gene expression compared to intact controls. Similarly, in injured GK tendons *Scx* and *Tnmd* gene expression was increased by 98% [p = 0.03] and 3-fold [p = 0.006] compared to those of intact GK rats (Fig 1A and 1B).

#### *Ngf*, *Bdnf*, *Tsp1* mRNA levels

At 2 weeks post-injury, the injured tendons of diabetic GK rats exhibited a mean 67% and 47% decrease in *Ngf* [p = 0.01] and *Tsp1* [p = 0.005] mRNA levels compared to the corresponding injured non-diabetic Wistar. In contrast, *Bdnf* gene expression levels in injured diabetic GK tendons were not significantly different to those of the injured Wistar controls.

Interestingly, the intact tendons of diabetic GK rats exhibited a significant mean 66% and 36% decrease in *Ngf* [p = 0.02] and *Tsp1* [p = 0.009] mRNA levels respectively, compared to intact control Wistar rats. Again, no differences in *Bdnf* gene expression were detected between intact tendons of diabetic GK and the Wistar controls.

**Fig 2:**
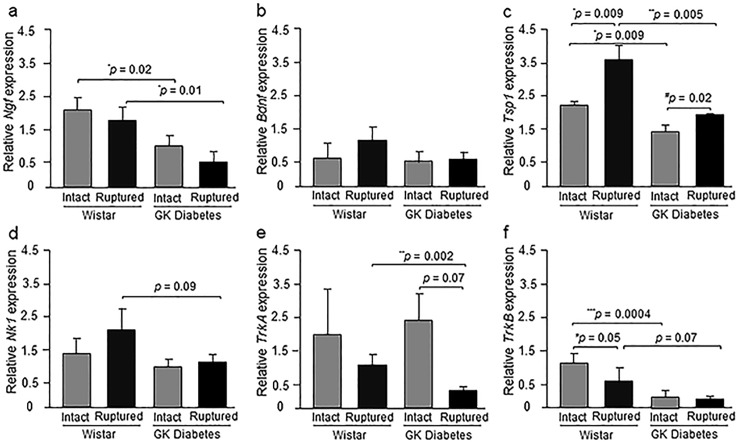
Relative expression of *Ngf*, *Bdnf*, *Tsp1*, *Nk1*, *Trka* and *Trkb* mRNA in the Achilles tendons. a) Relative gene expression of *Ngf* b) *Bdnf*, c) *Tsp1*, *d) Nk1*, e) *Trka* and f) *Trkb* in the intact and ruptured Achilles tendons of Wistar and diabetic GK rats at day 14 post-rupture. Results are expressed as mean ± SEM with n = 4 in Wistar groups and n = 5 in diabetic GK groups. *P ≤ 0.05, **P ≤ 0.005 and ***P ≤ 0.0005 compared to intact and ruptures Wistar and ^#^P ≤ 0.05 compared to intact GK group (ANOVA followed by Fisher’s protected least significant difference test).

Healing at 2 weeks post-injury led to significantly 39% up-regulated *Tsp1* mRNA levels [p = 0.009] in injured compared to intact tendons of Wistar rats. Similarly, in injured diabetic GK rat *Tsp1* mRNA levels were increased by 37% [p = 0.02] compared to corresponding intact diabetic tendons. Healing at 2 weeks did not significantly lead to alterations in the mRNA levels for *Ngf* or *Bdnf* within these groups (Fig 2A–2C).

#### *Trka*, *Trkb and Nk1* mRNA levels

At 2 week post-injury, injured tendons of diabetic GK rats exhibited 81% lower *Trka* mRNA levels [p = 0.002], and trends towards 66% and 41% lower *Trkb* [p = 0.069] and *Nk1* [p = 0.091] mRNA levels, respectively, compared to injured tendons of the Wistar controls. The intact diabetic GK tendons also exhibited 79% lower *Trkb* mRNA expression levels compared to the Wistar controls [p = 0.0004], but no detectable alterations in *Trka* and *Nk1* expression levels.

Within the Wistar controls, healing at 2 weeks post-injury led to a significant 66% down-regulation of *Trkb* mRNA levels compared to intact tendons of Wistar controls [p = 0.05]. In contrast, at 2 weeks post-injury within the healing diabetic tendons there was a trend for 28% lower *Trka* mRNA levels compared to intact GK tendons [p = 0.07] (Fig 2D–2F).

### Mediator protein and receptor expression in diabetic connective tissue healing

Generally, the immunohistochemical analysis of proteins detected specific localisations and confirmed the differences in gene expression observed between the groups with small variations (Fig 2A–2F).

#### NGF, BDNF, TSP1 expression

**Fig 3:**
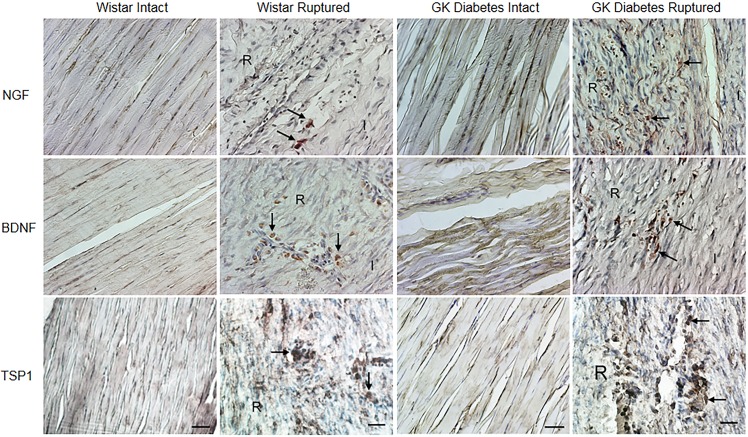
NGF, BDNF and TSP1 expression in Achilles tendon. Expression of NGF, BDNF and TSP1 in intact and ruptured tendon of Wistar and diabetic GK rats at day 14 post-rupture. Original magnification is 20x; bars = 100 μm. (I; Intact part of the tendon and R; Ruptured area).

In intact tendons of the diabetic GK and Wistar rats, strong NGF, BDNF and TSP1 immunoreactivity was observed in tenocytes, as well as in the matrix. However, no apparent differences were observed in intact tendons of the diabetic GK and Wistar rats. After injury, diabetic GK tendons exhibited much weaker NGF, BDNF and TSP1 immunoreactivity with fewer positively stained tenocytes compared to injured Wistar controls. In injured tendons of both Wistar and diabetic GK rats, NGF, BDNF and TSP1 positively stained cells were observed, especially localized to the injured areas and in the matrix (Fig 3).

#### NK1 and Trk receptor expression and distribution

**Fig 4:**
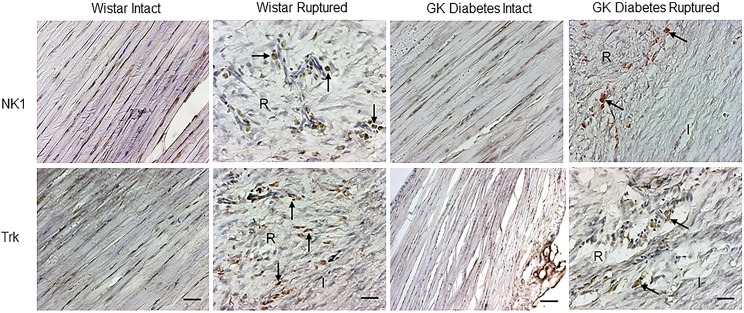
NK1 and Trk expression in Achilles tendon. Expression of NK1 and Trk in intact and ruptured tendon of Wistar and diabetic GK rats at day 14 post-rupture. Original magnification is 20x; bars = 100 μm. (I; Intact part of the tendon and R; Ruptured area).

In intact tendons of the diabetic GK and Wistar rats, no detectable expressional changes were observed in Trk and NK1 immunoreactivity. Overall, in injured tendons of both Wistar and diabetic GK rats, very weak NK1 and Trk immunoreactivity with fewer positively stained tenocytes were observed, especially in the callus, matrix and adjacent connective tissues (Fig 4).

### Discussion

This study demonstrates a compromised expression of neuro- and angiotrophic ligands (NGF, BDNF, TSP1), as well as receptor (TrkA, TrkB, NK1) genes and proteins in an animal model of type-II DM, findings which likely are contributing factors in altered healing in the diabetic state. Using the same animal model, we have previously demonstrated that the regenerative capability of diabetic tendon is compromised with corresponding impaired expressional changes in collagen I and III, matrix metalloproteinase-13 and various inflammatory and growth factor mediators [6, 19]. The findings of the present study suggest that altered neurotrophic and angiotrophic signaling may additionally be involved in regulating the impaired connective tissue healing and homeostasis associated with type-II diabetes.

This study assessed neuro- and angiotrophic signaling factors associated with connective tissue homeostasis and healing at two weeks post-injury, the transition phase between inflammatory and proliferative healing. The time point studied, two weeks, may be well suited to characterize the regulatory factors associated with neurogenesis and angiogenesis in rat Achilles tendon healing, since the peak of ingrowth of new nerves and blood vessels in this injury model has been observed during healing at weeks 2–6 [20]. Healing at 2 weeks post-rupture was confirmed by the up-regulated gene expression of tendon-related markers Scleraxis (*Scx*) and Tenomodulin (*Tnmd*). These results are in line with previous observations of increased *Scx* and *Tnmd* gene expression in injured tendons [21]. SCX is a transcription factor that is expressed in both tenogenic progenitors, as well as in tenocytes [22], and TNMD is a type II transmembrane protein specifically expressed in dense connective tissues such as tendons and ligaments [23].

Interestingly, the impaired neuro- and angiotrophic pathways as observed in the healing of diabetic connective tissue also appear to be affected in intact diabetic tendon homeostasis, although to a lesser extent. Thus, the lower expression levels detected for NGF, TSP1 and TrkB in the intact GK tendons as compared to the corresponding intact non-diabetic Wistar controls may reflect a broad range of potentially impaired neuro- and angiotrophic pathways also in the intact homeostasis of diabetic connective tissues. However, as compared to the healing diabetic tissue, which exhibited compromised TrkA expression, this was not observed in intact connective tissue.

The main results presented demonstrate that the expression of many of the neuro- and angiotrophic factors studied are depressed in the injured GK tendons, as compared to the corresponding injured non-diabetic Wistar controls. These findings suggest an important regulatory role for these factors in diabetic healing. The downregulation of NGF expression observed in diabetic healing tissue may contribute to impaired re-innervation and neuropeptide expression [24], blood flow [25], and angiogenesis [26]. In fact, exogenous NGF has been demonstrated to enhance ligament repair in rats [27]. NGF exerts its main effects via the TrkA receptor [10]. The present study also demonstrated lower expression of TrkA in injured GK tendons, as compared to the non-diabetic Wistar controls, indicating a likely combined NGF-TrkA induced impairment of diabetic connective tendon tissue healing.

The compromised NGF expression after injury in diabetic animals may also affect the production of different neuronal substances, e.g. Substance P (SP). After injury or inflammation, NGF can undergo retrograde transport in small nerves and after arriving in the dorsal root ganglia, stimulate the production of SP [28, 29]. Via its receptor NK1, newly produced and released SP can potentially stimulate angiogenesis and recruitment of stem cells to the injured area [12]. Hence, impaired NGF and NK1 expression in diabetic connective tissue healing may lead to decreased recruitment of stem cells and inadequate angiogenesis, outcomes that may be reflected by a more hypocellular and hypovascular diabetic tendon healing in the diabetic rats.

On the other hand, the decreased TSP1 expression observed in injured GK tendons as compared to the non-diabetic Wistar controls may suggest less inhibition of angiogenesis since TSP1 is known to inhibit angiogenesis. However, as TSP inhibits angiogenesis by specifically suppressing stimulation of NK1 [14], which also exhibited a trend towards lower expression; these effects may cancel each other out. Lower TSP1 levels in injured diabetic tendons as compared to the non-diabetic controls may also indicate decreased activation of transforming growth factor B (TFG-β). TFG-β has been demonstrated as an important growth factor in early connective tissue healing [30].

The unaltered BDNF mRNA levels in the injured diabetic GK tendons compared with the non-diabetic Wistar controls and the weak BDNF staining likely indicates that BDNF may not be a central regulatory molecule that is affected by diabetes, especially not at the two weeks healing time point. BDNF acts via its receptor TrkB and regulates neuroplasticity, and it is also upregulated following brain injury [31]. The finding of BDNF and TrkB in tendon, as confirmed by earlier studies [10, 32], as well as trends towards decreased TrkB expression in healing diabetic GK tendons compared with the non-diabetic controls, suggest a potential local regulatory role for this pathway in tendon connective tissue healing. Interestingly, tendon injury leads to increased mRNA levels for TrkB as observed in the non-diabetic Wistar tendons, whereas the corresponding diabetic GK rat tendons do not exhibit similar increases.

Hence, it may be concluded that the affected molecules in the neuro- and angiotrophic pathways in diabetes may have a more extensive impact on connective tissue repair than was previously observed [19], but also to some extent, may affect connective tissue homeostasis. In particular, tendon tissue homeostasis in diabetes may be vulnerable to the impact of repetitive loading events and/or combined with smaller injuries, which in turn may lead to degenerative changes as is seen in tendinopathy. In fact, tendinopathies are observed to occur to a greater extent in patients with diabetes than non-diabetics [2]. Indeed, we have previously reported lower stiffness both in the intact and healing diabetic tendons, smaller transverse area and lower stiffness associated with impaired structural organization of collagen fibers and reduced collagen I and III expression in injured diabetic tendons using same animal model [6]. Interestingly, both increased and decreased tendon stiffness in healing diabetic tendon are reported [33, 34], which probably represent diverse anatomical locations [34], differential healing phases of studied diabetic tendons or are specific to animal model used. In diabetic GK rats, the transverse area of the callus was not increased and the peak load was significantly reduced compared to intact tendons probably reflecting impaired healing [35].

Taken together our data suggest that the observed dysregulation of the neuro- and angiotrophic pathways in diabetes may lead to a less than ideal cascade of neuro-vascular mediators, leading to an accompanying neuropathy and microangiopathy contributing to compromised homeostasis and healing following an injury. We hypothesize that therapeutic modulation of NGF and TSP1 may potentially promote both re-innervation and angiogenesis in operated, injured or in degenerative tendon diseases in diabetic individuals.
